# Characterization of occupational exposures to cleaning products used for common cleaning tasks-a pilot study of hospital cleaners

**DOI:** 10.1186/1476-069X-8-11

**Published:** 2009-03-27

**Authors:** Anila Bello, Margaret M Quinn, Melissa J Perry, Donald K Milton

**Affiliations:** 1 Work Environment Department, University of Massachusetts Lowell, One Univ. Ave, Lowell, MA 01854, USA; 2 Department of Environmental Health, Harvard School of Public Health, 410 Park Drive, Boston, MA 02215, USA

## Abstract

**Background:**

In recent years, cleaning has been identified as an occupational risk because of an increased incidence of reported respiratory effects, such as asthma and asthma-like symptoms among cleaning workers. Due to the lack of systematic occupational hygiene analyses and workplace exposure data, it is not clear which cleaning-related exposures induce or aggravate asthma and other respiratory effects. Currently, there is a need for systematic evaluation of cleaning products ingredients and their exposures in the workplace. The objectives of this work were to: a) identify cleaning products' ingredients of concern with respect to respiratory and skin irritation and sensitization; and b) assess the potential for inhalation and dermal exposures to these ingredients during common cleaning tasks.

**Methods:**

We prioritized ingredients of concern in cleaning products commonly used in several hospitals in Massachusetts. Methods included workplace interviews, reviews of product Materials Safety Data Sheets and the scientific literature on adverse health effects to humans, reviews of physico-chemical properties of cleaning ingredients, and occupational hygiene observational analyses. Furthermore, the potential for exposure in the workplace was assessed by conducting qualitative assessment of airborne exposures and semi-quantitative assessment of dermal exposures.

**Results:**

Cleaning products used for common cleaning tasks were mixtures of many chemicals, including respiratory and dermal irritants and sensitizers. Examples of ingredients of concern include quaternary ammonium compounds, 2-butoxyethanol, and ethanolamines. Cleaning workers are at risk of acute and chronic inhalation exposures to volatile organic compounds (VOC) vapors and aerosols generated from product spraying, and dermal exposures mostly through hands.

**Conclusion:**

Cleaning products are mixtures of many chemical ingredients that may impact workers' health through air and dermal exposures. Because cleaning exposures are a function of product formulations and product application procedures, a combination of product evaluation with workplace exposure assessment is critical in developing strategies for protecting workers from cleaning hazards. Our task based assessment methods allowed classification of tasks in different exposure categories, a strategy that can be employed by epidemiological investigations related to cleaning. The methods presented here can be used by occupational and environmental health practitioners to identify intervention strategies.

## Background

Cleaning products have become an indispensable part of our modern lives. They are used on a daily basis in nearly all workplaces and homes. In recent years, cleaning has been identified as an occupational risk, because of an increased incidence of asthma and asthma-like symptoms among cleaning workers [1-7]. Adverse effects on skin, such as occupational hand dermatitis, have also been reported by few studies of hospital cleaning workers [8,9]. Results from epidemiological investigations support the hypothesis that exposure to cleaning products is related to the development and/or exacerbation of respiratory symptoms, including asthma [10-17]. The design of existing epidemiologic studies on cleaning has not allowed identification of agents responsible for asthma and other reported respiratory symptoms. This is related directly to the incomplete exposure assessment strategies carried out in these studies. Due to the lack of systematic occupational hygiene analyses and workplace exposure data, there is a need for systematic evaluation of cleaning products ingredients and their exposures in the workplace.

The main objective of this work was to characterize occupational exposures to cleaning products used for common cleaning tasks in hospitals. We have identified a set of cleaning products used for common cleaning tasks, evaluated the products' ingredients of concern in relation to respiratory and skin irritation and sensitization, and assessed the potential for inhalation and skin exposures during common cleaning tasks.

We selected hospitals to study cleaning exposures given the results of a recent surveillance report that identified cleaning products as one of the leading causes of occupational asthma among health care workers [3]. Additionally, we hypothesized that hospital cleaning workers may be at higher exposure risk compared to other groups of cleaners because: a) hospitals use a wide range of products including disinfectants, a toxicologically important group of chemicals in cleaning products[1]; and b) the frequency of the cleaning activities has increased rapidly in hospitals in order to ensure compliance with existing regulations and guidelines for protecting patients and workers from infectious diseases[18].

This paper reports data on potentially hazardous ingredients comprising products used everyday for common cleaning tasks in hospitals and identifies cleaning tasks that are associated with higher potential for exposures during product applications. The results of this work can be useful for epidemiologic studies for developing better exposure metrics to relate to health effects. Furthermore, the results are important for development of effective interventions in the workplace.

## Methods

### Identification of cleaning products and ingredients of concern

#### Evaluation of product lines and identification of chemical ingredients

Information on major products lines and brand names of cleaning products was obtained by interviewing environmental services representatives of six hospitals in Eastern Massachusetts. Hospitals selected represent a full range of in-patient and outpatient services and were located in several cities. They included three large sized urban teaching hospitals, one medium sized urban hospital, and two medium sized suburban hospitals.

We conducted interviews of cleaning workers and performed and observational surveys to identify the products used daily and the associated cleaning tasks. Material Safety Data Sheets (MSDSs) of the products were collected on site or obtained by the manufacturers' web sites. MSDSs of both the concentrated form and ready to use (RTU) form of all products were reviewed. The concentrated forms were evaluated even when only the RTU form was actually used in order to identify ingredients of the mixture with concentrations of less than 1% by weight, that are not reported from the MSDSs of RTU products. Information collected from MSDSs included hazardous ingredients as listed, their concentration in the mixture and chemical abstract services (CAS) numbers.

#### Determination of ingredients of concern in cleaning products

A list of chemical ingredients identified from MSDSs was created. Because cleaners were mixtures of many ingredients, a set of criteria was developed to prioritize ingredients for further exposure assessment evaluation. An ingredient was considered to be of concern if: 1) it occurred frequently in multiple cleaning products, 2) it was likely to cause respiratory and skin irritation and sensitization, 3) it occurred at higher concentrations compared to other ingredients in the product, or 4) had higher potential to become airborne compared to other mixture ingredients. First, a frequency analysis allowed identification of chemical ingredients that occurred at least three times in different products. Among them, all potential sensitizers were prioritized despite their concentration percentage in the product. Irritant ingredients were further prioritized based on their exposure potential during product application in the workplace (using criteria 3 and 4).

Potential health effects of identified ingredients, together with their physical-chemical properties were researched through literature review and online search of Toxnet's Hazardous Substances Data Bank (HSDB) and ChemIDplus [19], the ACGIH 2008 TLVs and BEIs booklet [20], and the NIOSH pocket guide to chemical hazards [21]. Applying the previously defined criteria, we prioritized ingredients among the large number of ingredient comprising cleaning mixtures. These ingredients were in the center of our further exposure assessment evaluation.

### Assessment of the potential for inhalation and dermal exposures

#### Identification of common cleaning tasks

Industrial hygiene worksite observation, interviews with workers, and videotaping of cleaning tasks were performed in three hospitals. Observations and interviews were performed for several hours while the workers were performing the tasks. Process flow charts were developed to identify cleaning tasks, which were used as a unit of exposure analysis. A "task" was defined as a cleaning activity that required application of one single product. Examples of common cleaning tasks performed include floor cleaning, mirror cleaning, toilet bowl cleaning, counter cleaning and floor finishing tasks.

#### Qualitative assessment of inhalation exposures

Potential inhalation exposures to ingredients of concern were assessed for each of the cleaning tasks identified. That was done qualitatively by taking into account both product formulations and task performance. Product formulation impacts directly the exposure intensity, depending on the volatility and concentration of ingredient in the product. Data on ingredients' volatility and concentrations were collected through literature searches and MSDSs review, respectively. Volatile organic compounds (VOC) were defined as compounds with boiling point between 0 – 400°C [1]. Boiling points were used relatively to assess the potential for inhalation exposures from different products; for example products that contain volatile ingredients with lower boiling points were considered to generate higher VOC exposures relative to others. Data on product application procedures (such as spraying vs. mopping), task duration and frequency were collected through workplace observations, videotaping and interviews of workers. Qualitative categorization of exposure intensity into low, medium and high, in combinations with task duration and frequency allowed classification of cleaning tasks in three potential inhalation exposure categories: low, medium, and high.

#### Semi-qualitative assessment of dermal exposures

The Dermal Exposure Assessment Method (DREAM), a validated semi-quantitative method for assessing dermal exposures, was applied to assess the potential for dermal exposure from common cleaning tasks [22-24]. The DREAM method is based on the conceptual model developed by Schneider that considers three major mechanisms by which the contaminant can contact the skin: emission, deposition and transfer[25]. Emission is the transport of substances from primary sources to the skin in the form of vapors or particles that can happen through splashing and spilling, for example. Deposition is the transport from air to the skin and it is dependent on the deposition velocity, concentration of the chemical in air and area of the skin contact. Transfer is the transport of substances by direct contact with skin for example from contaminated working tools [22].

The DREAM method has two major parts. The first part, the *inventory*, includes a structured questionnaire designed to collect data on six levels: company, department, agent, job, tasks and exposure module. The second part, *evaluation*, involves assessmentof the potential dermal exposure for each task using the following equation:

(A) ***Skin-P***_***TASK ***_= ***Σ Skin-P***_***BP***_

where: Skin-P_TASK _= Potential dermal exposure/task

Skin-P_BP _= Potential dermal exposure/body part

The potential skin exposure for each task is estimated as sum of potential skin exposure for 9 body parts: head, hands, upper arms, lower arms, torso front, torso back, lower body part, lower legs, and feet. The potential exposure for each body part is estimated as sum of the three major exposure routes: emission, deposition and transfer as follows:

(B) ***Skin-P***_***BP ***_= ***E***_***BP ***_+ ***D***_***BP ***_+ ***T***_***BP***_

where: E_BP _= exposure/body part through emission

D_BP _= exposure/body part through deposition

T_BP _= exposure/body part through transfer

The exposure potential for each route is estimated using the following equations:(C)

where: P = exposure probability

I = exposure intensity

E_I _= intrinsic emission

ER = exposure route factor

The main elements of the last set equations (C) are the probability (P) and the intensity (I) of exposure. The probability is assigned a value of 0, 1, 3, or 10 based on the frequency of the occurrence of exposure route. The intensity is also categorized in four categories and assigned values between 0, 1, 3, or 10. The intensity of emission and deposition is defined as amount of the agent on clothing and for transfer is defined as contaminated level of the surface. Another element in these equations is the "intrinsic emission" that accounts for physical and chemical properties of agents. For more details on how the intensity, probability and intrinsic emission values are assigned the reader can refer to the DREAM method[22].

Using these equations, we estimated the potential total body skin exposure per task (Skin-*P*_*TASK*_) for a set of common cleaning tasks: toilet bowl cleaning, sink cleaning, mirror cleaning, floor cleaning with traditional method and floor cleaning with microfiber mops. In the paper we present step by step estimations for two cleaning tasks. Based on the value of the Skin-*P*_*TASK*_, cleaning tasks were classified in different exposure categories as defined by DREAM: "no exposure", "low exposure", "moderate exposure", and "extremely high exposure". Additionally, body parts with the highest potential for dermal exposures were identified by comparing the DREAM estimates for different body parts.

## Results

### Ingredients of concern in cleaning products

The major product lines used for common cleaning tasks included general purpose cleaners, glass cleaners, washroom cleaners, and floor finishing products. Examples of products and their ingredients are given in Additional file 1. A list of chemical ingredients identified from MSDSs is given in Additional file 2. The most frequent ingredients (that occurred in more than three products) are highlighted in bold in Additional file 2.

Ingredients of concern identified based on the previously expanded criteria, included: quaternary ammonium chlorides or "quats", glycol ethers such as 2-butoxyethanol, ethanolamine, several alcohols such as benzyl alcohol, ammonia and several phenols. Additional file 3 presents a summary of ingredients' chemical and physical properties, health effects of their inhalation and dermal exposures, and the purpose of the application in cleaning products.

### Description of common cleaning tasks in hospitals

Common cleaning tasks identified included: preparation of cleaning solutions, floor cleaning, window cleaning, mirror cleaning, toilet bowl cleaning, sink cleaning, and floor finishing tasks (buffing, waxing and stripping).

#### Preparation of cleaning solutions

Cleaning solutions were prepared in the preparation room and were later transported to each floor using a cart. In most of the cases, solutions were prepared using an automated dispensing system. Concentrated cleaning products were diluted to the ready to use (RTU) form at a certain dilution rate. The dilution rate differed from one product to another; for example the dilution rate was higher for floor cleaners (rate = 3 gallon/min) than for glass cleaners (rate = 1 gallon/min). Only floor finishing products such as floor strippers were prepared by manual mixing.

#### Floor cleaning tasks

Two methods of floor cleaning were observed: a) wet mop cleaning and b) microfiber mop cleaning. The traditional method involved dipping the mop into a bucket filled with cleaning solution. The second involved the use of the microfiber cloths that were soaked by hand in cleaning solution, used attached to a handle, and send to laundry after one room was cleaned. Floor cleaning was performed daily and its duration varied by the size of the room. For example, patient room cleaning required about 5–10 minutes and hallway floor cleaning required several hours.

#### Window/mirror/glass cleaning tasks

During these tasks the product was sprayed and then wiped with paper towels. The frequency of window cleaning was lower compared to other tasks. Windows were cleaned as needed and mostly in the main areas or hallways with glass doors. Bathroom mirrors were cleaned daily using glass cleaners.

#### Bathroom cleaning tasks

Bathroom cleaning involved several cleaning tasks such as: sink cleaning, mirror cleaning, toilet bowl cleaning, and floor cleaning and required application of many products, specific for each task. For mirror and sink cleaning the product was sprayed and wiped with paper towels. During toilet bowl cleaning the product was sprayed into the toilet bowl, followed by brushing with a toilet cleaning brush. In general, bathrooms were cleaned two times per day. The average cleaning time varied from 10–15 minutes.

#### Floor finishing tasks (stripping, waxing, buffing)

During stripping the floor stripper was applied and left to reside on the floor for about 10 minutes. Then the old floor finish and the residue of the stripper were removed by using a stripping and a wet vacuum machine. Floor waxing was performed after stripping by mopping the protective coat on the floor. After waxing the floor was left for about 20–40 minutes to dry, depending on the indoor air temperature and humidity. Fans were usually used to speed up this drying process. Floor stripping was performed twice a year and in cases when floors were worn or scratched. Floor buffing was needed more frequently, and was performed by spraying the solution and finishing the localized area with a buffing machine.

#### Patient room cleaning

**Figure 1:**
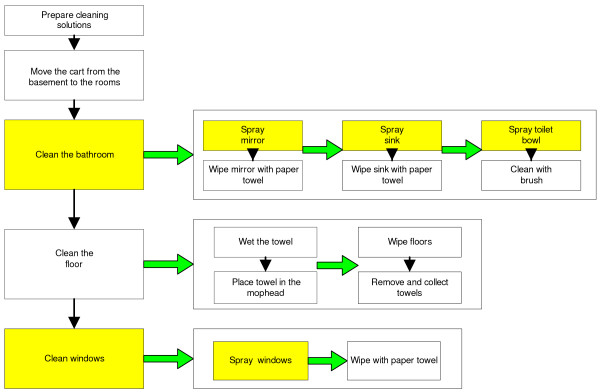
**Process flow diagram of the tasks performed for patient room cleaning**. The shaded boxes indicate cleaning tasks/steps with higher potential for inhalation exposure.

Patient room cleaning involved combination of several cleaning tasks, such as floor, counters and bathroom cleaning tasks. An example of a patient room cleaning flow chart is given in Figure 1. The workers were responsible for cleaning a certain number of patient rooms (in one case, 22 rooms) during the work shift.

### Inhalation exposure potential

Qualitative exposure assessment of inhalation exposures resulted in classification of cleaning tasks into three major exposure groups: low, medium, and high exposures.

#### Low exposure category

Tasks classified in this exposure category include floor cleaning tasks. Floor cleaning generates low concentrations of VOC in the air, mainly because floor products were more diluted compared to other products. Because quaternary ammonium compounds, an important group of chemicals of concern in floor cleaning products, are not volatile chemicals, the potential for their inhalation during floor cleaning is low. Additionally, because floor cleaning does not involve product spraying, the risk of inhalation to aerosol particles is low. Despite their longer duration compared to other tasks, considering their lower exposure intensity, floor cleaning tasks can be classified in the low inhalation exposure category.

#### Medium exposure category

Tasks classified in this exposure category include: window and mirror cleaning, sink cleaning, counter cleaning, and toilet bowl cleaning. The potential for inhalation exposures during these tasks is higher compared to floor cleaning tasks because: a) the intensity of VOCs of concern in the air is higher due to higher concentrations of volatile ingredients in the diluted products and b) product spraying may facilitate exposures to aerosols and other non-volatile ingredients, such as quats, commonly found in products used for these tasks. Workers performing these tasks are continuously exposed to VOCs and aerosols during the workday.

#### High exposure category

We classified in this category "the combination tasks", which include patient room and bathroom cleaning tasks. Due to the continuous application of many products one after another, the potential for inhalation exposures can be higher compared to when the tasks are performed separately. The shadowed boxes in Figure 1 show tasks that potentially generate higher airborne exposures. Because these tasks are done in small volume environments, it is possible that airborne VOC can increase rapidly in a short period of time exposing the worker to inhalation risks.

The other group of tasks classified in this category includes floor finishing tasks, such as stripping, waxing, and buffing. The potential for inhalation exposures from these tasks is higher compared to other tasks because: a) the airborne exposure intensity is higher due to higher VOC concentrations in the bulk product; b) they include specific activities such as the use of stripping and buffing machines, which can facilitate dust and particle re-suspension in the air that can potentially be inhaled; and c) the application of fans to speed up floor drying increases the intensity of exposures to VOC. Quantitative exposure assessment is necessary to evaluate the risk of particle inhalation during floor finishing tasks. Although less frequent overall, these tasks may contribute to high acute exposure levels that can be related to irritation mechanisms of asthma and other respiratory symptoms among cleaning workers.

### Dermal exposure potential

**Figure 2:**
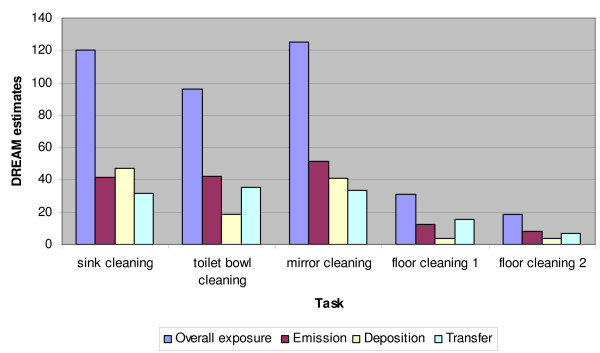
**Potential dermal exposure for five cleaning tasks**. Contribution of emission deposition and transfer to the overall potential body exposure is shown in addition to the overall potential for the total body dermal exposure.

**Figure 3:**
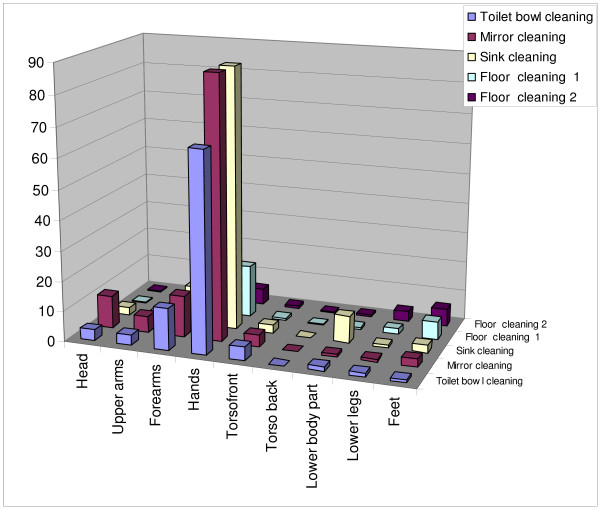
**Potential skin exposure for different body parts during different cleaning tasks**. Floor cleaning 1 represents floor cleaning with microfiber mops and floor cleaning 2 represents floor cleaning with the traditional mop & bucket method.

Two *examples *of step by step estimations of potential skin exposures for mirror and floor cleaning are given in Additional files 4 and 5. The results of DREAM estimates for five cleaning tasks are presented in a graph given in Figure 2. This graph presents the total body potential skin exposure for five tasks along with contribution of three exposure routes emission, deposition and transfer for each task. The results of individual body part contribution to the potential total body dermal exposure/task for the five cleaning task evaluated is presented in Figure 3.

According to the DREAM categories, cleaning tasks create moderate (such as in floor cleaning tasks) and high potential for dermal exposure (such as in mirror/window cleaning, sink cleaning and toilet bowl cleaning tasks). We identified the relative contribution of three dermal exposure routes for different tasks as shown in Figure 2. As seen from this graph, the "emission" route contributes more to the overall exposure compared to "transfer" and "deposition" routes during mirror and toilet bowl cleaning. One possible explanation to this finding is related to the spraying activities that generate liquid particles with aerodynamic diameter >100 μm that potentially reach the skin. In the case of sink cleaning, the "emission" is lower because the potential for aerosol particles to reach the head and upper body parts here is lower compared to mirror and toilet bowl spraying. Transfer contributed more during floor cleaning, probably due to the continuous hand contact with the mop handle contaminated with cleaning solution. Overall, floor cleaning tasks were associated with the lowest potential for dermal exposures. Hands were identified as having the highest potential for dermal exposure for most of the tasks. Forearms were at the next highest risk of exposure during sink, toilet bowl & mirror cleaning while for floor cleaning, feet and lower legs were most prone to exposure.

## Discussion

This study investigated exposures generated from common cleaning tasks in hospitals by considering both the product formulations and exposure potential to ingredients of concern during product applications. This work shows that:

### 1. Cleaning products are complex mixtures of many chemicals

The chemical ingredients identified in the products included disinfectants, surfactants, solvents, and fragrances. These ingredients are representative of different chemical classes such as ethers, alcohols, amines, acids and have a very wide range of volatilities and other chemical properties. The same chemical ingredients we have identified here have been previously reported by several studies [1,26,27].

When investigating ingredients using product MSDSs, health and safety professionals should review not only MSDSs of concentrated product forms, but also the ready to use forms. We found that many ingredients reported in the concentrated form were missing in the RTU form, because MSDSs are required to list only ingredients at concentrations greater than 1% in the product. This is important for identifying ingredients that are sensitizers in the workplace; given the fact that sensitization may occur even at trace concentrations.

One important finding is related to the high frequency of use of disinfectants among different product groups. Disinfectants are added to the cleaning products with the main goal to destroy microbial life. On the other hand, cleaning is done with the goal of mechanically removing the surface contaminants. An important question that can be raised is: Can disinfectants achieve their goal if they are applied in combination cleaner-disinfectant product? In order for disinfection to be effective, it should follow surface cleaning and the disinfectant should reside on the surface for about 10–15 minutes after application[28]. In the case of combination product (cleaner-disinfectant) application, these procedures can not be followed. The effectiveness of disinfectants used for common cleaning activities has been questioned in the literature [29-31]. Although the evidence to date is minimal, repeated application of disinfectants may increase the risk of microbial resistance, which will require the use of stronger disinfectants in order to be destroyed [32,33]. Given: 1) the uncertainty of disinfectant effectiveness in cleaning public areas, 2) the risk of inducing bacteria resistance, and 3) the health concerns related to the use of disinfectants, it is critical to further evaluate disinfectants' effectiveness for common cleaning activities and to develop workplace strategies for preventing workers from exposures to disinfectants. Such strategies may include purchasing of green cleaning products, identification of the areas where disinfection is needed, and following the necessary disinfection procedures in the cases when disinfection is necessary.

### 2. There is evidence of exposures to respiratory and dermal irritants and sensitizers from cleaning products

2-Butoxyethanol (2-BE), a glycol ether with boiling point (BP) of 168°C, was commonly used in cleaning products including glass/window cleaners, carpet cleaners and other surface cleaners[36]. Indoor exposures to its vapors at a concentration threshold of 2 ppm (10 mg/m3) and above may result in sensory irritation [34]. The OSHA permissible exposure limit (PEL) is 50 ppm for 8 h time weighed average (TWA), the ACGIH threshold limit value (TLV) is 20 ppm (8 h TWA) and the NIOSH recommended exposures limit (REL) is 5 ppm (10 hour exposure). There is a skin designation for 2-BE from both OSHA and NIOSH, indicating that 2-BE can be absorbed through the skin. The presence of 2-BE in cleaning products has been reported by several studies [35,36]. Concentrations of 2-BE in the air generated during window cleaning reported by Vincent 1993 ranges from 0.1–7.33 ppm, lower than existing occupational standards. The study suggested that dermal exposure may be the most important exposure route in the workplace[37]. Because 2-BE was one of the most frequent solvents in our products and had the highest concentrations in the bulk products, it is important to further assess its workplace exposures. Quantitative workplace investigations are necessary to measure the degree of exposure intensity and relationship with irritation symptoms reported among cleaning workers.

Quaternary ammonium compounds, or quats, were widely used in many of the products investigated. Quats have been identified by Nielsen 2007 as one of the indoor agents that may promote development of airway allergy[27]. In his review, Nielsen summarizes the evidence from animal and humans studies that relates quats exposures with allergy-promoting effects. He concludes that the mechanism of asthma from quats remains unknown. One important consideration in the investigation of asthma symptoms from quats is the understanding of exposure routes in the workplace. Although several case reports identify an asthma-quats exposure relationship, they lack the clarification on how exposure had occurred [2,5,6]. In the second case report of a study by Purohit 2000, the nurse developed symptoms after entering the room that was cleaned with a surface cleaning product that contained benzalkonium chloride[5]. Because the nurse was not involved in cleaning activities the most probable exposure route would be inhalation and not through skin. A study by Vincent 2006 showed non-detectable levels of quats in the hospital indoor air [38]. Inhalation to quats may happen in two ways: 1) by inhaling aerosolized liquid particles generated during product application, or 2) by inhaling quats absorbed into the dust particles that are re-suspended in the air. Further quantitative workplace investigations of inhalation and dermal exposures will provide important evidence for understanding actual exposure routes for quats.

Mono-ethanolamine, used as surfactant, was found in most of the product types investigated, with exception of the floor cleaners. It has a boiling point of 171°C and dissolves very well in water. Exposures to its vapors can irritate the nose, throat, and lungs, causing coughing, wheezing and shortness of breath. The OSHA PEL for mono-ethanolamine is 3 ppm and the ACGIH 15 min short term exposure limit (STEL) is 6 ppm. Exposure to mono-ethanolamines from cleaning agents have been related to occupational asthma [4]. To understand the exposure response relationship and the mechanism of asthma, it is necessary to investigate exposure patterns in the workplace including short term peak exposures. Dermal exposure assessment should be considered in further workplace exposure assessment strategies, given the concern that mono-ethanolamine can be absorbed through the skin [19].

Fragrances were used commonly in bathroom cleaners. Exposure to fragrances is a topic of special interest because they may cause secondary emissions due to reactions of the primary exposures with oxidizers present in indoor air (e.g. terpenes, a family of chemicals common in fragrances, reacting with ozone in indoor air) [39]. These reactions can produce secondary gaseous and aerosol ultrafine particles that may be responsible for the airway irritation symptoms [40,41]. Very recently, Wolkoff (2008) found that gaseous products generated from ozone-limonene reactions are causative of acute irritation effects measured in a mice bioassay[42]. Another important consideration related to fragrance use in cleaning products is the presence of odor during and after cleaning. Even at low concentrations, the presence of compounds with low odor thresholds may cause perceived respiratory irritation because of odor annoyance[41]. Furthermore, this is a topic of special interest due to the reported sensitization effects associated with the fragrance use [43].

Surprisingly, bleach was not used in any of these products compared to findings from other studies, which found that bleach can be responsible for asthma symptoms among domestic cleaners [14,44].

### 3. Cleaning tasks generate airborne exposures

Volatile compounds identified in cleaning products covered a wide range of volatilities, from highly volatile ingredients such as ammonia (BP = -33°C) and isopropyl alcohol (BP = 82°C) and relatively less volatile ingredients such as 2-butoxyethanol (BP = 168°C) and mono-ethanolamine (BP = 171°C). The highest intensity of VOC exposures in the workplace is expected during the use of floor strippers and general purpose cleaners because they contain the highest concentrations of VOCs in the bulk. Inhalation exposure to aerosol particles of volatile and non-volatile ingredients can be facilitated during product spraying. The worst exposure scenarios can happen when several cleaning tasks are performed in small and poorly ventilated spaces, such as bathrooms.

Hazardous exposures related to cleaning products are an important public health concern because these exposures impact not only cleaning workers, but also other occupants in the building. Data from laboratory studies indicate a two phase decay of the air concentrations in the room. The first phase decay happens very fast (in the first 10 minutes) and the second phase decay happens slowly (about 1–2 hours for the air concentrations to reach the background level). Furthermore, experimental studies have shown that some compounds such as glycol ethers are released slowly from the surfaces. This creates potential for exposure of other occupants in the building, hours after the cleaning activities are performed [35,36]. The intensity of exposures after the completion of cleaning has not been investigated in field studies. In a follow up study we will conduct quantitative assessment airborne exposures during cleaning and will provide evidence on the exposure levels after cleaning.

### 4. Cleaning tasks create potential for dermal exposures

Application of the DREAM method in this pilot study confirmed the applicability of this method for categorization of cleaning tasks in different dermal exposure categories. Exposure categories identified included two groups: "high" (for sink, mirror and toilet bowl cleaning) and "moderate" (for floor cleaning with two different methods) exposures. The difference between these two groups of tasks may reflect the product applications procedures, such as spraying (typical for the first group of tasks) versus mopping (the second group of tasks). The DREAM method did not find differences within tasks that involve spraying and within the tasks that do not employ spraying. Both floor cleaning methods were in the same exposure category, even though there were important changes in the cleaning procedures (such as dipping the hands into cleaning solution during the microfiber mop method). This limitation has also been observed by the DREAM authors, who recommend the method is most appropriate for detecting high contrast exposure levels[23]. Furthermore, for all cleaning tasks, DREAM identified hands as the body part at higher potential for dermal exposure compared to other body parts. Overall, our results suggest that dermal exposure prevention should focus mostly on hands and the activities that involve product spraying.

The DREAM observational analyses applied here showed that dermal exposure can be an important route for chemicals in the body. Recent literature suggests that some chemical ingredients, such as isocyanates, may be able to penetrate the skin and cause systemic respiratory effects [45]. Dermal exposure should be evaluated in future studies of health effects of cleaning.

### 5. This work showed that cleaning exposures are a function of the way that tasks are performed, as well as product formulations

A comprehensive approach to exposure prevention will account for the method with which a product is applied and the task requirements, as well as assessing of the chemical ingredients and implementation of safer alternatives to cleaning products.

### Limitations

The results of this work are based on a small number of products. While we selected a few representative hospitals, it is possible that other products with additional ingredients are used elsewhere.

This study does not address the lack of quantitative data in the literature regarding the concentrations of cleaning compounds in workplace air. Quantitative characterization of exposures would better identify activities that produce the highest exposure, important for control measures. This work serves as preparation for a detailed quantitative assessment of airborne exposures from cleaning tasks.

## Conclusion

This study found that cleaning products are mixtures of many chemical ingredients of concern that may impact worker health through air and dermal exposures. Because cleaning exposures are a function of both product formulations and product application procedures, a combination of product evaluation and workplace exposure data is necessary to develop strategies for protecting workers from cleaning hazards. The task based assessment conducted here allowed classification of cleaning tasks in different exposure categories, a strategy that can be employed by epidemiological investigations of the impact of cleaning on health. The methods presented here can also be used by occupational and environmental health practitioners to identify workplace interventions for improving health.

## Abbreviations

MSDSs: materials safety data sheets; RTU: ready to use; CAS: chemical abstract services; HSDB: hazardous substances data bank; ACGIH: American Conference of Governmental Industrial Hygienists; OSHA: Occupational Safety and Health Administration; NIOSH: National Institute for Occupational Safety and Health; TLV: Threshold Limit Values; REL: Recommended Exposure Limit; PEL: Permissible Exposure Levels; BEI: Biological Exposure Indexes; VOC: volatile organic compounds; DREAM: dermal exposure assessment method; BP: boiling point; 2-BE: 2-butoxyethanol.

## Competing interests

The authors declare that they have no competing interests.

## Authors' contributions

AB led the design of the research, drafted the paper, carried out the workplace interviews and observations, and performed exposure assessment analyses. MMQ co-led the design of the research, guided paper writing, facilitated workplace connections and conducted paper revisions. MJP and DKM provided critical input on criteria for product selection, representative data collection in the workplace, adapting the DREAM method for cleaning tasks and contributed to paper revisions. All authors approved the final manuscript.

## Supplementary Material

Additional file 1 **Table S1. **Examples of cleaning products used and their chemical ingredients. Click here for file

Additional file 2 **Table S2.** Chemical ingredients reported in the cleaning product material safety data sheets (MSDSs). Click here for file

Additional file 3 **Table S3.** The most hazardous ingredients in cleaning products investigated. Click here for file

Additional file 4 **Table S4.** Potential dermal exposure estimates for floor cleaning (using microfiber mops). Click here for file

Additional file 5 **Table S5.** Potential dermal exposure for mirror cleaning. Click here for file
