# Supplementary material for: Characterization of occupational exposures to cleaning products used for common cleaning tasks-a pilot study of hospital cleaners
Source: Environ Health. 2009 Mar 27;8:11. doi: 10.1186/1476-069X-8-11 (PMC2678109; doi:10.1186/1476-069X-8-11)
Supplement: Additional file 1 — Table S1. Examples of cleaning products used and their chemical ingredients. [file 1476-069X-8-11-S1.doc]

Table 1: Examples of cleaning products used and their chemical ingredients.

| Product name | Chemical ingredient name | **Chemical Abstract Services (CAS) number** | **Ingredient product concentration ^a)^**  **(% by weight)** |
| --- | --- | --- | --- |
| **Floor Cleaners** |  |  |  |
| Neutral cleaner (Concentrate) | Water  Hydroxyalkyl amine oxides  Isopropyl alcohol  2-Ethyl-hexyloxyethanol  Fragrance | 7732-18-5  68478-65-9  67-63-0  1559-35-9  unknown | 40-70  15-40  5-10  5-10  <2 |
| Neutral cleaner ^b)^  (Ready to use) | Water  Hydroxyalkyl amine oxides | 7732-18-5  68478-65-9 | > 98  < 1 |
| Dimension II disinfectant non-alkaline cleaner (Concentrate) | Didecyl dimethyl ammonium chloride  N-Alkyl dimethyl benzyl ammonium chloride  Octyl dimethyl amine oxide  Ethylenediamine tetra acetic acid  Ethyl alcohol | 7173-51-5  8001-54-5  2605-78-9  60-00-4  64-17-5 | 10-25  5-7  3-5  3-5  3-5 |
| Dimension II disinfectant non-alkaline cleaner  (Ready to use) | Didecyl dimethyl ammonium chloride  N-Alkyl dimethyl benzyl ammonium chloride | 7173-51-5  8001-54-5 | <0.1  <0.1 |
| Dimension III ^TM^  one step disinfectant cleaner  (Concentrate) | N-Alkyl dimethyl benzyl ammonium chloride  Didecyl dimethyl ammonium chloride | 68391-01-5  7173-51-5 | 8.190  8.704 |
| Dimension III ^TM^  one step disinfectant cleaner  (Ready to use) | N-Alkyl dimethyl benzyl ammonium chloride  Didecyl dimethyl ammonium chloride  Water | 68391-01-5  7173-51-5  1132-18-5 | <1  <1  >99 |
| **Floor Care Products** |  |  |  |
| Floor stripper  (Concentrate) | Benzyl alcohol  Ethanolamine  Benzenesulfonic acid derivative | 100-51-6  141-43-5  70191-75-2 | 40-70  30-60  1-5 |
| Floor stripper  (Ready to use) | Water  Benzyl alcohol  Ethanolamine | 7732-18-5  100-51-6  141-43-5 | >90  1-5  1-5 |
| Pro strip concentrated  (Concentrate) | Sodium xylene sulphonate  Benzyl alcohol  2-Buthoxyethanol  Mono ethanolamine  Water | 1300-72-7  100-51-6  11176-2  141-43-5  7732-18-5 | 1-5  5-10  10-30  10-30  60-100 |
| Over drive spray buff  (Ready to use) | Diethylene glycol mono ethyl ether | 111-90-0 | 1-5 |
| Low profile floor stripper  (Concentrate) | Mono ethanolamine  Sodium hydroxide  Diethylene glycol butyl ether  Benzyl alcohol | 141-43-5  1310-73-2  112-34-5  100-51-6 | 5-10  1-5  1-5  10-25 |
| Gemstar Stratus  Floor care product  (Ready to use) | 2-(2-ethoxyethoxy) ethanol | 111-90-0 | 3-7 |
| Bright speed floor care product  (Ready to Use) | 2-Aminoethanol  Ethoxylated alcohol  Xylene sulfonic acid sodium salt | 141-43-5  68439-46-3  1300-72-7 | 5-10  -  1-5 |
| **General purpose cleaners** |  |  |  |
| Speedball heavy duty spray cleaner  (Concentrate ) | 2-Buthoxyethanol  Ethanolamine  Diethyl pthalate | 111-76-2  141-43-5  84-66-2 | 25-40  10-25  0.1-1.5 |
| Speedball heavy duty spray cleaner  (Ready to use) | 2-Buthoxyethanol  Ethanolamine | 111-76-2  141-43-5 | 5-7  1-3 |
| Heavy duty multi surface cleaner  (Concentrate) | 1-Methoxy 2- propanol  Water  Ethanolamine  Hydroxyalkyl amine oxides  Isopropyl alcohol  Dodecyl benzene sulfonic acid  2-Ethyl –hexyloxyethanol  2-methoxy-1-propanol | 107-98-2  7732-18-5  141-43-5  68478-65-9  67-63-0  27176-87-0  1559-35-9  1589-47-5 | 40-70  10-30  10-30  5-10  1-5  1-5  1-5  1-5 |
| Heavy duty multi surface cleaner  (Ready to use) | Water  1-Methoxy 2- propanol  Ethanolamine | 7732-18-5  107-98-2  141-43-5 | >90  1-5  1-5 |
| Quat disinfectant cleaner (Concentrate ) | Water  Alkyl dimethyl benzyl ammonium chloride  Octyl decyldimethyl ammonium chloride  Nonylphenoxypoly ethanol  Ethyl alcohol  Didecyl dimethyl ammonium chloride  Ehtylene diamine tetracetic acid tetrasodium salt  Dioctyl dimethyl ammonium chloride  Sodium metasilicate  Fragrance  Dye | 7732-18-5  68424-85-1  32426-11-2  9016-45-9  64-17-5  7173-51-5  64-02-8  5538-94-3  6834-92-0  Unknown  Unknown | 70  8.7  6.5  5-10  1-5  3.9  1-5  2.6  1-5  0.5-1.5  <.1 |
| Quat disinfectant cleaner (Ready to use) | Water  Alkyl dimethyl benzyl ammonium chloride  Octyl decyldimethyl ammonium chloride  Didecyl dimethyl ammonium chloride  Dioctyl dimethyl ammonium chloride | 7732-18-5  68424-85-1  32426-11-2  7173-51-5  5538-94-3 | >99  <0.1  <0.1  <0.1  <0.1 |
| GP forward general purpose cleaner  (Concentrate ) | Alcohol ethoxylates  Propylene glycol methyl ether | 68439-46-3  107-98-2 | 5-10  5-10 |
| GP forward general purpose cleaner  (Ready to use) | Alcohol ethoxylates  Water | 68439-46-3  7732-18-5 | <1  60-100 |
| General purpose cleaner (Concentrate ) | Water  Hydroxyalkyl amine oxides  Isopropyl alcohol  2-ethyl-hexyloxyethanol | 7732-18-5  68478-65-9  67-63-0  1559-35-9 | 40-70  15-40  5-10  5-10 |
| General purpose cleaner  (Ready to use) | Water  Hydroxyalkyl amine oxides | 7732-18-5  68478-65-9 | >98  <1 |
| 20 Hot springs heavy duty general purpose cleaner  (Concentrate) | Sodium metasilicate  Linear primary alcohol ethoxylate  Amphoteric surfactant  Tetrasodium ethylenediamine tetraacetate  Quaternary ammonium compounds | 6834-92-0  34398-01-1  na  64-02-8  na | 7-10  5-7  1-3  1-3  1-1.5 |
| 20 Hot Springs heavy duty general purpose cleaner (Ready to use) | Sodium metasilicate | 6834-92-0 | 0.1- 0.5 |
| Quest 256 neutral disinfectant cleaner  (Concentrate) | Octyl decyl methyl amine  N-alkyl dimethyl benzyl ammonium chloride  Di-n alkyl dimethyl ammonium chloride  Alkyl (C12-16) dimethyl amine oxide  Tri sodium salt of NTA  Ethyl alcohol | 22020-14-0  68424-85-1  68424-95-3  68439-70-3  5064-31-3  64-17-5 | 3.2  8.7  6.5  3.2  0.1-1.5  1-5 |
| Quest 256 neutral disinfectant cleaner  (Ready to use) | Alkyl dimethyl benzyl ammonium chloride  Octyl decyl dimethyl ammonium chloride  Dioctyl dimethyl ammonium chloride  Didecyl dimethyl ammonium chloride | 68424-85-1  68424-95-3  5538-94-3  7173-51-5 | <0.1  <0.1  <0.1  <0.1 |
| Phenolic disinfectant cleaner ( Concentrate) | Water  Ethylene glycol  0-benzyl-p-chlorophenol  0-phenylphenol  Sodium hydroxide | 7732-18-5  107-21-1  120-32-1  90-43-7  1310-73-2 | 60-90  1-10  9.5  9.5  1-5 |
| **Bathroom cleaners** |  |  |  |
| Bath mate acid free disinfectant washroom  (Concentrate) | 2-Buthoxyethanol  Secondary alcohol ethoxylate  Ethanolamine  Fragrance  Tetrasodium ethylenediamine tetra acetate  N-Alkyl dimethyl benzyl ammonium chloride  Didecyl dimethyl ammonium chloride | 111-76-2  68131-40-8  141-43-5  NA  64-02-8  68-424-85-1  7173-51-5 | 25-40  10-25  7-10  3-5  1-1.5  0.25-1  <0.1 |
| Bath mate acid free disinfectant washroom  (Ready to use) | 2-Buthoxyethanol | 111-76-2 | 1-3 % |
| Bathroom cleaner (Concentrate) | 1-octyl -2-pyrolidinone  Hydroxyacetic acid  Water  Malic acid  Quaternary ammonium chlorides  Fragrance  Ethyl alcohol | 2687-94-7  79-14-1  7732-18-5  6915-15-7  68424-95-3  Unknown  64-17-5 | 10-30  10-30  10-30  10-30  5-10  1-5  1-5 |
| Bathroom cleaner  (Ready to use) | Water  1-Octyl -2-pyrolidinone  Hydroxyacetic acid  Malic acid | 7732-18-5  2687-94-7  79-14-1  6915-15-7 | > 95  <1  <1  <1 |
| Non-acid disinfectant bathroom cleaner (Concentrate) | Water  Nonyl phenoxypoly ethanol  Alkyl dimethyl benzyl ammonium chloride  Octyl decyl dimethyl ammonium chloride  Ethyl alcohol  Tetrasodium ethylene diamines tetra acetate  Dioctyl dimethyl ammonium chloride  Didecyl dimethyl ammonium chloride | 7732-18-5  9016-45-9  68424-85-1  68424-95-3  64-17-5  64-02-8  5538-94-3  7173-51-5 | 60-90  1-5  4.3  3.2  1-5  1-5  1.6  1.6 |
| Non-acid disinfectant bathroom cleaner  (Ready to use) | Water  Nonyl phenoxypoly ethanol  Aryl tri alkyl ammonium compounds  Quaternary ammonium compounds | 7732-18-5  9016-45-9  68424-85-1  68424-95-3 | > 99  <0.1  <0.1  <0.1 |
| Non acid bathroom cleaner (Concentrate ) | Water  Carboxy imidazolinium salt  Fragrance | 7732-18-5  13039-35-5  unknown | 60-90  15-40  1-5 |
| Non acid bathroom cleaner (Ready to use) | Water  Carboxy imidazolinium salt  Fragrance | 7732-18-5  13039-35-5  unknown | >95  <1  <1 |
| Bath guard acid free disinfectant bathroom cleaner  (Ready to use) | Alkyl dimethyl benzyl ammonium-chloride  Dipropylene glycol methyl ether | 139-07-1  34590-94-8 | 0.3  1-3 |
| **Glass Cleaners** |  |  |  |
| Brand Glass Cleaner (Concentrate ) | 1-propoxy 2- propanol  2-propoxy-1- propanol  Propylene glycol  Dipropylene glycol  Dipropylene glycol n-propyl ether  Laureth 6 carboxylic acid  Fragrance  Ethanolamine | 1569-01-3  10215-30-2  57-55-6  25265-71-8  29911-27-1  157707-83-0  Trade Secret  141-43-5 | 60-100  1-10  1-10  < 5  <5  1-5  1-5  0.5-1.5 |
| Brand Glass Cleaner (Ready to use) | Water  1-propoxy propanol | 7732-18-5  1569-01-3 | > 95  0.5-1.5 |
| 2 Look Glass Cleaner (Concentrate) | 2- Butoxyethanol  Propylene glycol methyl ether  Alcohol ethoxy sulfate  Ammonium hydroxide  Tetrasodium ethylenediamine tetraacetate  Ethyl alcohol | 111-76-2  107-98-2  NA  1336-21-6  64-02-8  64-17-5 | 25-40  5-7  5-7  3-5  1-3  0.25-1.0 |
| 2 Look Glass Cleaner  (Ready to use) | Ammonium hydroxide | 1336-21-6 | 0.1-0.5 |
| 3 Glisten Glass and Surface Cleaner  (Concentrate) | 2-Butoxyethanol  Ammonia  Ethylene glycol | 111-76-2  7664-41-7  107-21-1 | 40-60  3-5  0.1-0.5 |
| 3 Glisten Glass and Surface Cleaner  (Ready to use) | 2-Butoxyethanol  Ammonia | 111-76-2  1336-21-6 | 1-3  0.25-1.0 |

^a)^ Ingredient product concentrations were obtained from product Material Safety Data Sheets (MSDSs).

^b)^ Shadowed boxes represent product ingredients listed from MSDSs of ready to use products.
