# Supplementary material for: Characterization of occupational exposures to cleaning products used for common cleaning tasks-a pilot study of hospital cleaners
Source: Environ Health. 2009 Mar 27;8:11. doi: 10.1186/1476-069X-8-11 (PMC2678109; doi:10.1186/1476-069X-8-11)
Supplement: Additional file 2 — Table S2. Chemical ingredients reported in the cleaning product material safety data sheets (MSDSs). [file 1476-069X-8-11-S2.doc]

**Table 2: Chemical ingredients reported in the cleaning product material safety data sheets (MSDSs)^a^.**

| *1* | *1-Methyl 1-4 (1- Methylethenanal) Cyclohexen Emulsion* | *29* | Ethylene glycol |
| --- | --- | --- | --- |
| *2* | *1-Octyl -2-pyrolidinone* | *30* | *Hydroxyacetic acid* |
| *3* | ***2-Butoxyethanol*** | *31* | ***Hydroxyalkyl amine oxides*** |
| ***4*** | *2-Ethyl –hexyloxyethanol* | *32* | *Isobutane* |
| *5* | *2-Methoxy-1-propanol* | *33* | ***Isopropyl alcohol*** |
| *6* | ***Alcohol ethoxylates*** | *34* | *Laureth 6 carboxylic acid* |
| *7* | *Aliphatic petroleum distillates* | *35* | *Linear primary alcohol ethoxylate* |
| *8* | ***Alkyl dimethyl benzyl ammonium chloride*** | *36* | *Malic acid* |
| *9* | ***Ammonium hydroxide*** | *37* | *Mono isopropanol amine* |
| *10* | *Amphoteric surfactant* | *38* | *N-Alkyl dimethyl ethyl benzyl ammonium chloride* |
| *11* | *Benzenesulfonic acid derivative* | *39* | *Nonionic surfactant* |
| *12* | ***Benzyl alcohol*** | *40* | *Nonyl phenol ethoxylate* |
| *13* | *Butane* | *41* | *Nonyl phenoxypoly ethanol* |
| *14* | *Carboxy imidazolinium salt* | *42* | *Octyl decyl dimethyl ammonium chloride* |
| *15* | *Calcium carbonate* | *43* | *Octyl dimethyl amine oxide* |
| *16* | *Citric acid* | *44* | ***Ortho-benzyl-para chloro-phenol*** |
| *17* | ***Didecyl dimethyl ammonium chloride*** | *45* | ***Ortho-phenyl-phenol*** |
| *18* | *Diethyl phthalate* | *46* | *Para-tertiary-amyl phenol* |
| *19* | *Di-ethylene glycol mono methyl ether* | *47* | *Polyethylene emulsion* |
| *20* | *Dioctyl dimethyl ammonium chloride* | *48* | ***Propylene glycol methyl ether*** |
| *21* | *Dipropylene glycol butoxy ether* | *49* | *Quaternary ammonium chlorides* |
| *22* | ***Dipropylene glycol methyl ether*** | *50* | *Secondary alcohol ethoxylate* |
| *23* | *Dodecyl benzene sulfonic acid* | *51* | *Silica, quartz* |
| *24* | *Dye* | *52* | *Sodium hydroxide* |
| *25* | ***Ethyl alcohol*** | *53* | *Sodium metasilicate* |
| *26* | ***Ethanolamine or (*** *2-Aminoethanol)* | *54* | *Sodium xylene sulphonate* |
| *27* | *Ethylene diamine tetra acetic acid* | *55* | ***Tetrasodium ethylene diamine tetraacetate*** |
| *28* | ***Fragrance*** | *56* | *Tri butoxy ethyl phosphate* |

^a)^*Ingredients with an appearing in 3 or more products are highlighted in bold.*
