# Supplementary material for: Characterization of occupational exposures to cleaning products used for common cleaning tasks-a pilot study of hospital cleaners
Source: Environ Health. 2009 Mar 27;8:11. doi: 10.1186/1476-069X-8-11 (PMC2678109; doi:10.1186/1476-069X-8-11)
Supplement: Additional file 3 — Table S3. The most hazardous ingredients in cleaning products investigated. [file 1476-069X-8-11-S3.doc]

**Table 3: The most hazardous ingredients in cleaning products investigated.**

| **Name** | **Chemical formula** | **Physicochemical properties** | **Inhalation exposure** | **Skin exposure** | **Sensitization** | **Purpose of use in cleaning products** |
| --- | --- | --- | --- | --- | --- | --- |
| **Quaternary ammonium compounds** | Examples :    Di-decyl di-methyl ammonium chloride    N Alkyl dimethyl benzyl ammonium chloride  (benzalkonium chloride) | Quaternary ammonium compounds are salts that are soluble in water and alcohol [1]. | Because they are not volatile compounds inhalation exposures can happen through products’ aerosolization. Commonly used solutions can cause nose and throat irritation. Benzalkonium chloride is a serve eye irritant [1]. | Benzalkonium chloride is a primary skin irritant in solutions of less than 10%. Exposures to quats may cause allergic reactions to skin [2]. | Limited evidence implicates quats in the development of allergic responses and occupational asthma [3, 4]. Exposures to benzalkonium chloride have been associated with combined respiratory and dermal hypersensitivity[2]. | Used as a low-level disinfectant. High level of disinfection is achieved if different quats and alcohols are mixed.  Mostly used in bathroom, floor, and general purpose cleaners. |
| **Glycol ethers**  **2-Buthoxyethanol** | Examples :    2-Butoxyethanol (2-BE) | 2-BE boiling point (BP):168 ^O^C. | 2-BE vapors are irritants to eyes and respiratory tract [5].  PEL^a^ :50 ppm  TLV^b^ -20ppm [6]  RD_50_^c^-2824ppm [7]  Class A3 : Confirmed animal carcinogen with unknown relevance to humans [6]. | 2-BE is a skin irritant.  It is absorbed in the body through skin[8]. Dermal exposure is an important exposure route[9] [11].  BEI: butoxy-acetic acid in urine: 200 mg/g creatinine [6, 10]. | No evidence of sensitization was found in the literature. | Used as solvent in cleaning products to dissolve fatty substances. Mostly used in glass, general purpose cleaners, and floor care products. |
| **Ethanolamine** | Mono-ethanol amine | BP: 170.8 ^0^C | Breathing its vapors can irritate the nose, throat and lungs, causing coughing, wheezing and shortness in breath [1].  PEL: 3 ppm  TLV: 3ppm | It is a skin irritant and it can be absorbed through the skin. Can cause skin sensitization[1]. | Exposures to ethanolamine has been linked to occupational asthma [11]. | Used as surfactant  in cleaning products. Used in floor care products, general purpose, glass and bathroom cleaners. |
| **Alcohols** | Examples :    Benzyl alcohol      Isopropyl alcohol | BP: 205 ^0^ C  BP: 82.5 ^0^ C | Benzyl alcohol :  Workplace Env. Exposure level, TWA is 10ppm.  Isopropyl alcohol: Highly volatile. Irritant to eyes, and the upper respiratory tract. Prolonged exposure may cause lung damage [1].  PEL : 400 ppm  TLV : 200 ppm  RD_50_ :5000ppm [7] | Slightly irritant to skin. Dermal exposure is a potential exposure route[1].  Repeated skin exposure can cause itching, redness, rash, drying and cracking.  Irritates and burns the skin [1]. | Benzyl alcohol has been reported as a contact allergen in cleaning products [12, 13]. | Are used as solvents and disinfectants in cleaning products. |
| **Ammonia** | NH_3_ | Found in the aqueous solutions on the form of ammonium hydroxide.  (BP): -33.5 ^0^ C. | Highly irritant. Inhalation to its vapors can irritate the nose, throat, lungs causing wheezing and shortness of breath. Prolonged exposure can cause bronchitis[1, 14].  PEL :50ppm ; TLV=25ppm  RD_50_-303 pm | Even at low concentrations is extremely irritating to the skin. Repeated skin contact can cause dryness, itching and redness. Can penetrate the skin[1]. | No evidence of sensitization was found. | Used in glass cleaners. |
| **Phenols/chlorinated phenols** | Ortho benzyl para chlorophenol (OBPC)    Ortho phenyl phenol (OPP) | OBPC  BP: 160-162 ^0^C  OPP  BP: 286 ^0^C | Irritant to eyes and respiratory tract when inhaled [15]. | Moderate skin irritant. Occupational exposures may happen mostly through dermal contact [1]. | Sensitivity potential reported from animal studies [16]. | Phenols are used as disinfectants in cleaning products |

1. PEL=Permissible Exposure Limit occupational standard enforced by Occupational Health and Safety administration (OSHA)
2. TLV= Threshold Limit Value Standard recommended by American Conference of Governmental Industrial Hygienists (ACGIH)
3. RD_50_= Concentrations of airborne chemicals at which a 50% decrease in respiratory rate can occur (developed by animal studies).

**Table 3 Reference list**

1. National Library of Medicine: **Hazardous Substances Data Bank and ChemIDplus.** In *Environmental Health and Toxicology, Toxicological Data Network (Toxnet)*: National Institutes of Health (NIH), Bethesda MD.

2. Bernstein JA, Stauder T, Bernstein DI, Bernstein IL: **A combined respiratory and cutaneous hypersensitivity syndrome induced by work exposure to quaternary amines.** *J Allergy Clin Immunol* 1994, **94:**257-259.

3. Purohit A, Kopferschmitt-Kubler MC, Moreau C, Popin E, Blaumeiser M, Pauli G: **Quaternary ammonium compounds and occupational asthma.** *Int Arch Occup Environ Health* 2000, **73:**423-427.

4. Burge PS, Richardson MN: **Occupational asthma due to indirect exposure to lauryl dimethyl benzyl ammonium chloride used in a floor cleaner.** *Thorax* 1994, **49:**842-843.

5. Starek A, Szabla J: **[Ethylene glycol alkyl ethers--the substances noxious to health].** *Med Pr* 2008, **59:**179-185.

6. American Conference of Governmental Industrial Hygienist (ACGIH): *Threshold Limit Values for Chemical Substances and Physical Agents and Biological Exposure Indices* Cincinnati , OH 2008.

7. Schaper M: **Development of a database for sensory irritants and its use in establishing occupational exposure limits.** *American Industrial Hygiene Association Journal* 1993, **54:**488-544.

8. Jakasa I, Mohammadi N, Kruse J, Kezic S: **Percutaneous absorption of neat and aqueous solutions of 2-butoxyethanol in volunteers.** *Int Arch Occup Environ Health* 2004, **77:**79-84. Epub 2003 Aug 2012.

9. Vincent R, Cicolella A, Surba I, Reieger B, Poirot P, Pierre F: **Occupational exposure to 2-butoxyethanol for workers using window cleaning agents.** *Applied Occupational and Environmental Hygiene* 1993, **8:**580-586.

10. Franks SJ, Spendiff MK, Cocker J, Loizou GD: **Physiologically based pharmacokinetic modelling of human exposure to 2-butoxyethanol.** *Toxicology Letters* 2006, **162:**164-173.

11. Savonius B, Keskinen H, Tuppurainen M, Kanerva L: **Occupational asthma caused by ethanolamines.** *Allergy* 1994, **49:**877-881.

12. Flyvholm MA: **Contact allergens in registered chemical products.** *Contact Dermatitis* 1991, **25:**49-56.

13. Flyvholm MA: **Contact allergens in registered cleaning agents for industrial and household use.** *Br J Ind Med* 1993, **50:**1043-1050.

14. Basil AA, Hafiz OA, Seifeddin GB, Albar AA: **Pulmonary function of workers exposed to ammonia.** *Int J Occup Environ Health* 2001, **7:**19-22.

15. Stouten H, Bessems JG: **Toxicological profile for o-benzyl-p-chlorophenol.** *Journal of Applied Toxicology* 1998, **18:**271-279.

16. Stern ML, Brown TA, Brown RD, Munson AE: **Contact hypersensitivity response to o-benzyl-p-chlorophenol in mice.** *Drug Chem Toxicol* 1991, **14:**231-242.
