# Supplementary material for: Characterization of occupational exposures to cleaning products used for common cleaning tasks-a pilot study of hospital cleaners
Source: Environ Health. 2009 Mar 27;8:11. doi: 10.1186/1476-069X-8-11 (PMC2678109; doi:10.1186/1476-069X-8-11)
Supplement: Additional file 4 — Table S4. Potential dermal exposure estimates for floor cleaning (using microfiber mops). [file 1476-069X-8-11-S4.doc]

| **Body part** | **Emission** | **Deposition** | **Transfer** | **Intrinsic emission (E)** | ***Skin-P_BP_*** |
| --- | --- | --- | --- | --- | --- |
|  | **E_BP_= P_E.BP_ * I_E.BP_ * ER_E_ * E** | **D_BP_= P_D.BP_ * I_D.BP_ * ER_D_*E** | **T_BP_= P_T.BP_ * I_T.BP_ * ER_T_*E** | **PS*C*EV*V** |  |
| Head | E_HE_= 0 | D_HE_=3*1*1*0.09= 0.27 | T_HE_=0 | E=1*0.3*0.3*1 | 0.27 |
| Upper arms | E_UA_= 0 | D_UA_=3*1*1*0.09= 0.27 | T_UA_=0 | 0.09 | 0.27 |
| Lower arms | E_FA_ =1*1*3 *0.09 =0.27 | D_FA_ =3*1*1*0.09= 0.27 | T_FA_ =3*10*1 *0.09=2.7 | 0.09 | 3.24 |
| Hands | E_HA_= 3*10*3* 0.09=8.1 | D_HA_ = 3*1*1*0.09= 0.27 | T_HA_ = 10*10*1*0.09= 9 | 0.09 | **17.37** |
| Torso front | E_TF_ = 1*1*3*0.09=0.27 | D_TF_ = 3*1*1*0.09= 0.27 | T_TF_ = 1*3*1*0.09=0.27 | 0.09 | 0.81 |
| Torso back | E_TB_ = 0 | D_TB_ = 3*1*1*0.09= 0.27 | T_TB_ = 0 | 0.09 | 0.27 |
| Lower body part | E_LB_ = 1*1*3*0.09=0.27 | D_LB_ =3*1*1*0.09= 0.27 | T_LB_ = 1*3*1*0.09=0.27 | 0.09 | 0.81 |
| Lower legs | E_LL_ =3*1*3*0.09=0.81 | D_LL_ = 3*3*1*0.09=0.81 | T_LL_ = 1*3*1*0.09=0.27 | 0.09 | 1.89 |
| Feet | E_FE_  = 3*3*3*0.09=2.43 | D_FE_  = 3*3*1*0.09=0.81 | T_FE_  = 3*10*1*0.09= 2.7 | 0.09 | 5.94 |
| ***Skin-P_task_*** *for total body* | 12.15 | 3.51 | **15.21** |  | **30.87** |

**Table 4: Potential dermal exposure estimates for floor cleaning (using microfiber mops)^a^.**

*^a)^ The main ingredients of concern in floor cleaning products are quaternary ammonium compounds*

*Main activities involved dipping the towel into the cleaning solution and mopping*
