# Supplementary material for: Characterization of occupational exposures to cleaning products used for common cleaning tasks-a pilot study of hospital cleaners
Source: Environ Health. 2009 Mar 27;8:11. doi: 10.1186/1476-069X-8-11 (PMC2678109; doi:10.1186/1476-069X-8-11)
Supplement: Additional file 5 — Table S5. Potential dermal exposure for mirror cleaning. [file 1476-069X-8-11-S5.doc]

**Table 5: Potential dermal exposure for mirror cleaning ^a^.**

| **Body part** | **Emission** | **Deposition** | **Transfer** | **Intrinsic emission (E)** | ***Skin-P_BP_*** |
| --- | --- | --- | --- | --- | --- |
|  | **E_BP_= P_E.BP_ * I_E.BP_ * ER_E_ * E** | **D_BP_= P_D.BP_ * I_D.BP_ * ER_D_*E** | **T_BP_= P_T.BP_ * I_T.BP_ * ER_T_*E** | **PS*C*EV*V** |  |
| Head | E_HE_=3*3*3*0.3= 8.1 | D_HE_=3*3*1*0.3= 2.7 | T_HE_=0 | E=1*0.3*1*1=0.3 | 10.8 |
| Upper arms | E_UA_=1*3*3*0.3=2.7 | D_UA_=3*3*1*0.3=2.7 | T_UA_=0 | 0.3 | 5.4 |
| Lower arms | E_FA_ =3*3*3 *0.3=8.1 | D_FA_ =3*3*1*0.3=2.7 | T_FA_ =1*10*1 *0.3=3 | 0.3 | 13.8 |
| Hands | E_HA_ = 3*10*3 *0.3=27 | D_HA_ = 10*10*1*0.3=30 | T_HA_ = 10*10*1*0.3=30 | 0.3 | **87** |
| Torso front | E_TF_ = 1*1*3*E=0.9 | D_TF_ = 3*3*1*0.3=2.7 | T_TF_ = 1*1*1*0.3=0.3 | 0.3 | 3.9 |
| Torso back | E_TB_ = 0 | D_TB_ = 0 | T_TB_ = 0 | 0.3 | 0 |
| Lower body part | E_LB_ = 1*1*3*E=0.9 | D_LB_ = 0 | T_LB_ = 0 | 0.3 | 0.9 |
| Lower legs | E_LL_ =1*1*3*E=0.9 | D_LL_ = 0 | T_LL_ = 0 | 0.3 | 0.9 |
| Feet | E_FE_  = 1*3*3*E=2.7 | D_FE_  = 0 | T_FE_  = 0 | 0.3 | 2.7 |
| ***Skin-P_task_*** *for total body* | **51.3** | 40.8 | 33.3 |  | **125.4** |

*^a)^ The main ingredient of concern in glass cleaning products is 2-butoxyethanol and main cleaning activities involve spraying and wiping*
